# Feasibility of a Group Version of the Lifestyle-Integrated Functional Exercise Program

**DOI:** 10.1093/geroni/igaa057.606

**Published:** 2020-12-16

**Authors:** Carl-Philipp Jansen, Corinna Nerz, Sarah Labudek, Jochen Klenk, Lindy Clemson, Clemens Becker, Michael Schwenk

**Affiliations:** 1 Heidelberg University, Karlsruhe, Germany; 2 Robert-Bosch Hospital Stuttgart, Stuttgart, Germany; 3 Heidelberg University, Heidelberg, Germany; 4 Unversity of Sydney, Sydney, Australia

## Abstract

In a randomised noninferiority trial, it is investigated whether a group version of the Lifestyle-integrated Functional Exercise program (gLiFE) is non-inferior to the individually delivered LiFE in terms of feasibility and effectiveness. While effectiveness evaluation is ongoing, feasibility results are already available. Participants (>70 years; confirmed fall risk) were randomized in either LiFE or gLiFE and participated in the same strength and balance exercises, however, based on different approaches of delivery. LiFE participants received seven home visits; gLiFE was delivered in seven group sessions. Feasibility was defined as willingness to participate, adherence to group/home visits, and drop-outs. Predictors for intention to participate were calculated using regression. N=310 participants were randomized to LiFE (n=156) or gLiFE (n=154). n=51 (16%) of the participants dropped out after baseline. Attendance analyses showed that when excluding drop-outs, 100% (iLiFE) and 88% (gLiFE) took part in at least 5 of the 7 meetings. Self-efficacy and outcome expectancies, but not risk perception, were predictors of the intention to participate (F(3,193)=24.84, p<.001). In this first study comparing a group-based LiFE format with the original LiFE, feasibility of both formats was shown in terms of high attendance and less drop-outs than expected in this target group. Compared to other studies involving group based training, compliance to intervention (defined as having absolved at least 5 sessions) was high in both formats. Lower attendance in gLiFE can be explained by inflexible scheduling as compared to making individual home visit appointments. Whether lower gLiFE adherence translates into lower effectiveness is currently analysed.

